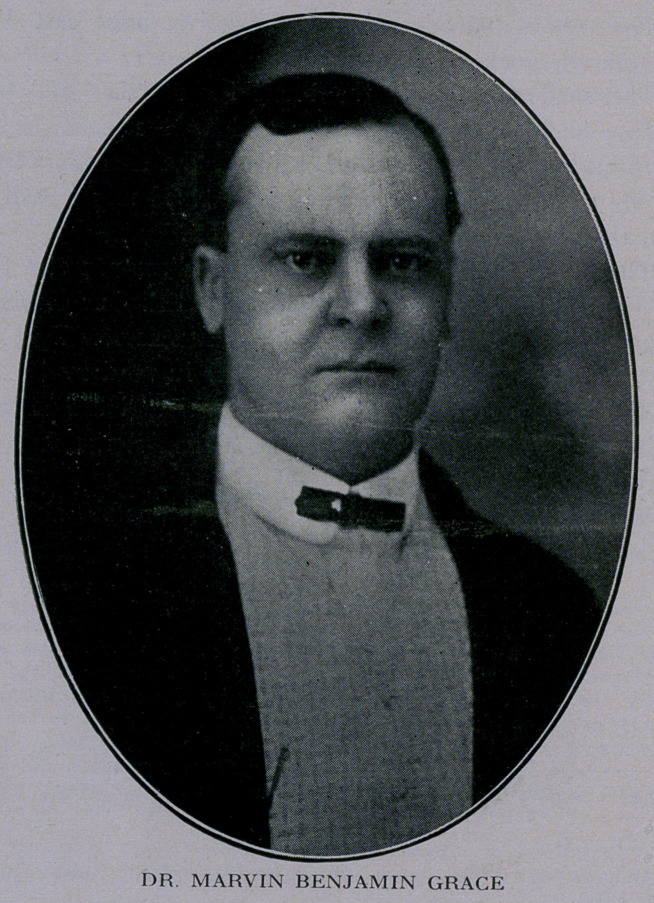# A Tribute to Dr. M. B. Grace

**Published:** 1915-09

**Authors:** 


DR. MARVIN BENJAMIN GRACE

### Dr. Marvin Benjamin Grace

Dr. Grace has left us; no more shall we see his erect form passing here and there through our council halls; no more shall we behold the light of friendship kindling in his splendid eyes, or feel the firm, warm grasp of his kindly hand. Our hearts are sad. His death came as a terrible shock to his many friends over the State. He was one of the finest men in the medical profession—clean, highminded, honorable, the soul of courtesy and chivalry; he had few equals and no superiors. Those who knew him best loved him most, and that is the highest tribute that could be paid to any man. We copy the following from the Seguin papers, as it expresses the sentiments of those who knew him most intimately:

### DR. MARVIN BENJAMIN GRACE.

Dr. Grace was born January 28, 1868, and died July 27, 1915, aged 47 years, 5 months and 23 days. He is survived, to mourn their loss, by his wife and children, Wysong, Gladys, Esther and Mildred, his mother, one brother and two sisters.

He was buried July 28, 1915, in Geronimo Cemetery by the Masons. Revs. J. T. Curry, of San Antonio, and James F. Pennybacker, Pastor of the First Methodist Church of Seguin, conducted the religious services at the residence. A large concourse of friends and relatives had gathered at the residence and cemetery.

Dr. Grace was a man much beloved by all. In his going away the community has lost a valued citizen, his profession an honored and efficient servant, the church a loyal and devoted member, the home a loving and ideal husband and father.

But such men as this do not die. The influence of their life is immortal, and the mantle thereof is constantly falling upon other shoulders. No doubt many in the community will be actuated to a larger and better life because of this man.

An old custom used to be: to come to the grave and deposit a sprig of evergreen, and say: "We release you from the duties of this life!" laboring under the idea that the spirit was given rest because of this release. At a burial, upon one occasion, a little boy deposited the evergreen in the grave, and said: "Papa, we sets you free, but you will be such a missin'!" So with our departed friend. We would not hold him back from the joys of that better life, but oh! he will be such a "missing." He sleeps well.—*Seguin Gazette,* July 30, 1915.

DR. MARVIN B. GRACE.

It is sad beyond the power of expression to think of Marvin B. Grace as dead. But, in doing so, we take occasion to give utterance to our appreciation of his virtues, and bear testimony to those high qualities in him that marked him in many respects as one of the leading men of his city and State.

It is peculiarly appropriate that his fellow members of the Masonic fraternity should perpetuate his memory because he was one of its most distinguished members and always gave it a place of unusual warmth in his affections.

It is proper and right that we should place upon record our estimate of his worth. His mind was exceedingly subtle, and his perceptible powers unusually and remarkably keen, and this made him the great physician that he was. He comprehended at a glance, and could make a diagnosis as if by intuition.

But superb as were his mental gifts, it was not this alone that made him great and gave him power such as few ever possessed. It was his great heart that endeared him to us all and made us love him and rejoice in his success.

True love is unmistakable in its manifestations. He who really and truly loves his fellows need not fear that they will fail to find it out. It will manifest itself, not in the arts and wiles of the hypocrite, but in a thousand ways which need not be premeditated, and cannot be misjudged or misunderstood.

He loved humanity, not in the abstract, but in the person of those members of it who came within reach of him. His love for his friends was not a mere sentiment, but a real passion, to which he gave expression in his never-tiring acts of devotion and his ceaseless efforts to aid them in every way and by every means that lay in his power. It was thus that he grappled his friends to him with hoops of steel and held them in a grasp which nothing could loosen.

But he is gone and we can only mourn his loss, and indulge the hope that the good he has done may live after him, and that even the sad bereavement of his death may help to bring his countrymen together as one in a union of fellowship and love.

*Resolved,* That in the death of our brother, Marvin B. Grace, our Masonic fraternity has lost one of its most honored and devoted members.

*Resolved,* That we tender to the bereaved family our sincere and heartfelt sympathy.

*Resolved,* That the city papers be requested to publish these proceedings and that a copy be spread upon the minutes of the Masonic fraternity.

JAS. GREENWOOD,
N. A. POTH,
H. E. SHORT,
Committee.

—*Seguin Bulletin,* August 4, 1915.

---

The Arlington Heights Female College of Fort Worth has been sold to Dr. W. F. Rountree and several other physicians and business men for the purpose of establishing a sanitarium for the treatment of nervous diseases. Dr. Rountree and his associates will constitute the faculty of the proposed sanitarium, which will have a special department for pellagra. The building is being thoroughly renovated and repaired.

---

Physicians are responsible for more than one-half of the drug fiends in the United States, according to a bulletin published by the public health service.

The bulletin is based on a study of cases which have come to the notice of the service since the Harrison anti-narcotic law went into effect. The study was made by Martin I. Wilbert, technical assistant in the hygienic laboratory.

At the outset Mr. Wilbert states that the number of drug victims in the United States is probably not much over 200,000. He denies that the passage of the Harrison law has caused any great suffering in the country or any crime wave such as was feared.

Mr. Wilbert comments on the deaths reported in Chicago, which were attributed to the difficulty of the unfortunates in obtaining narcotics after the law went into effect. He also refers to the marked increase in the number of drug victims admitted to Philadelphia hospitals.

These cases, according to Mr. Wilbert, in no way measured up to the predictions of persons who believed the law would cause great suffering and much crime.

Studies of the drug fiends indicated that more than one-half of them were started in the vice by physicians who prescribed either morphine, cocaine, or heroin while attending them in a professional capacity.

A large number of persons have become users of drugs on the advice of acquaintances who use them to relieve suffering, while an equal number picked up the habit through bad associates.

---

The discovery of a specific for cerebro-spinal meningitis was announced recently by Dr. Richard Bull, director of the bacteriological laboratory of the University of Melbourne. Dr. Bull stated that eucalyptus would destroy the germ.

The medical properties of the oil obtained from leaves of the eucalyptus tree have long been recognized. It has been used in treatment of microbic disease of the lungs and bronchi and employed as an antiseptic.

---

Dr. Alexis Carrel of the Rockefeller Institute for Medical Research and Dr. Henry D. Dakin of the Lister Institute have discovered, after exhaustive experiment at the Compiegne Military Hospital, what they claim to be the ideal antiseptic.

The most powerful antiseptic known to science is hypochlorite of lime, but its use is injurious to the tissues, owing to its acidity, and it does not keep. The two physicians have found these two defects are remedied, respectively, by the addition of carbonate of lime and boric acid. If applied in time the new antiseptic is said to make infection in wounds impossible.

---

The latest University bulletin is "A Model Health Code for Texas Cities," by Robert M. Jameson, Secretary of the Bureau of Municipal Research and Reference. The bulletin contains concrete suggestions to the cities of Texas, which, according to the preface, "may be followed, in whole or in part, as conditions seem to warrant." The ordinances have been collected from far and wide and represent the thought of many minds. The bulletin is distributed free through the University Bureau of Municipal Research.

---

Miss Winnie Morrison, daughter of Dr. and Mrs. M. M. Morrison, of Denison, proved herself a true heroine as well as an expert swimmer Wednesday morning when she rescued from almost certain death by drowning Emily Wiley of that city. Miss Morrison should have a Carnegie medal.

## Figures and Tables

**Figure f1:**